# Significance of intratissue estrogen concentration coupled with estrogen receptors levels in colorectal cancer prognosis

**DOI:** 10.18632/oncotarget.23309

**Published:** 2017-12-14

**Authors:** Agnieszka Anna Rawłuszko-Wieczorek, Łukasz Marczak, Nikodem Horst, Karolina Horbacka, Piotr Krokowicz, Paweł Piotr Jagodziński

**Affiliations:** ^1^ Department of Biochemistry and Molecular Biology, Poznań University of Medical Sciences, Poznan, Poland; ^2^ European Centre for Bioinformatics and Genomics, Institute of Bioorganic Chemistry, Polish Academy of Sciences, Poznan, Poland; ^3^ Department of General and Colorectal Surgery, Poznań University of Medical Sciences, Poznan, Poland

**Keywords:** estrogen, estrogen receptors, colorectal cancer

## Abstract

Dysregulation of estrogen related pathways is implicated colorectal cancer (CRC) development. However, significance of intratissue concentration of estrone (E1) and 17β-estradiol (E2) in relation to estrogen receptor (ESR) expression level was not addressed so far. Herein, we measured E1 and E2 intratissue concentration using liquid chromatography electrospray ionization tandem mass spectrometry (ESI LC/MS) and mRNA levels of ESR1 and ESR2 using RT-qPCR in cancerous and histopathologically unchanged tissue from 75 and 110 CRC patients, respectively. The obtained results were associated with clinicopathological factors, expression of estrogen dependent genes (*CTNNB1*, *CCND1*) and prognostic significance. We found no statistically significant differences in E1 or E2 concentration between cancerous tissue and histopathologically unchanged counterparts. Moreover, mRNA levels of ESR1 and ESR2 were significantly decreased in cancerous tissue compared with histopathologically unchanged (p=0.00001). Log rank analysis revealed no benefit of low E1 to E2 ratio, high E1, E2 concentration or ESR1, ESR2 mRNA level for patients’ overall (OS) and disease free survival (DFS). Interestingly, we have observed that patients with low ESR1 mRNA level coupled with low E1 intratissue concentration had a significant decrease in DFS compared with group of patients with high ESR1 mRNA level and high E1 concentration (HR=0.16, 95% CI 0.02-1.05; p=0.06). Furthermore, patients with low E1 concentration and low ESR1 transcript had significantly higher CTNNB1 and CCND1 mRNA level compare with subgroup with high level of both grouping factors. Our study indicates a potential value of estrogen intratissue concentration and its receptor expression level for CRC patients’ prognosis.

## INTRODUCTION

Even though multiple factors are involved in colorectal cancer (CRC) development the CRC occurs less frequently among women in all susceptibility groups [1]. Gender differences suggest potent role of steroid hormones. Premenopausal women have lower CRC incidence than age-matched men [2, 3]. Additionally, most of the prospective and retrospective studies showed an inverse relationship between the risk of CRC incidence and the use of hormone replacement therapy (HRT) by postmenopausal women [4–6]. Moreover, in animal studies, ovariectomized rats exposed to estrone had significantly reduced tumor growth [7] whereas ovariectomized Apc^Min/+^ mice had increased number of polyp formation [8].

Interestingly, metabolism of estrogen, including the synthesis of most biologically active form, 17β-estradiol (E2), may take place in peripheral tissues, including the large bowel. Estrone (E1) can be produced in extragonadal tissues from C19 precursors via the sulfatase or aromatase pathway [9]. Subsequently, E1 may be converted into E2 by 17β-hydroxysteroid dehydrogenases (HSD17βs) [9].

Further, estrogen manifest its cellular effect primarily by estrogen receptors ER-α and -β, encoded by *ESR1* and *ESR2* respectively [9]. Importantly, *ESR2* expression was inversely correlated with CRC progression and dKO of *Esr1* or *Esr2* in ovariectomized Apc^Min/+^ resulted in increased rate of tumor formation [10–13].

Despite of potential significance of estrogen metabolism within peripheral tissues, the concentrations of intratissue estrogens were only once investigated in CRC clinical samples. Moreover, CRC risk may differ depending on the expression level of ERs. Therefore, we measured E1 and E2 levels in cancerous and histopathologically unchanged tissue from 75 CRC patients’ samples as well ESR1 and ESR2 mRNA levels from 110 patients. Next we addressed the question if expression level of ER in relation to matched intratissue estrogen concentration affects patients’ clinical outcome.

## RESULTS

### Intratumoral concentrations of E1 and E2 in cancerous and adjacent histopathologically unchanged colorectal tissue

**Figure 1:**
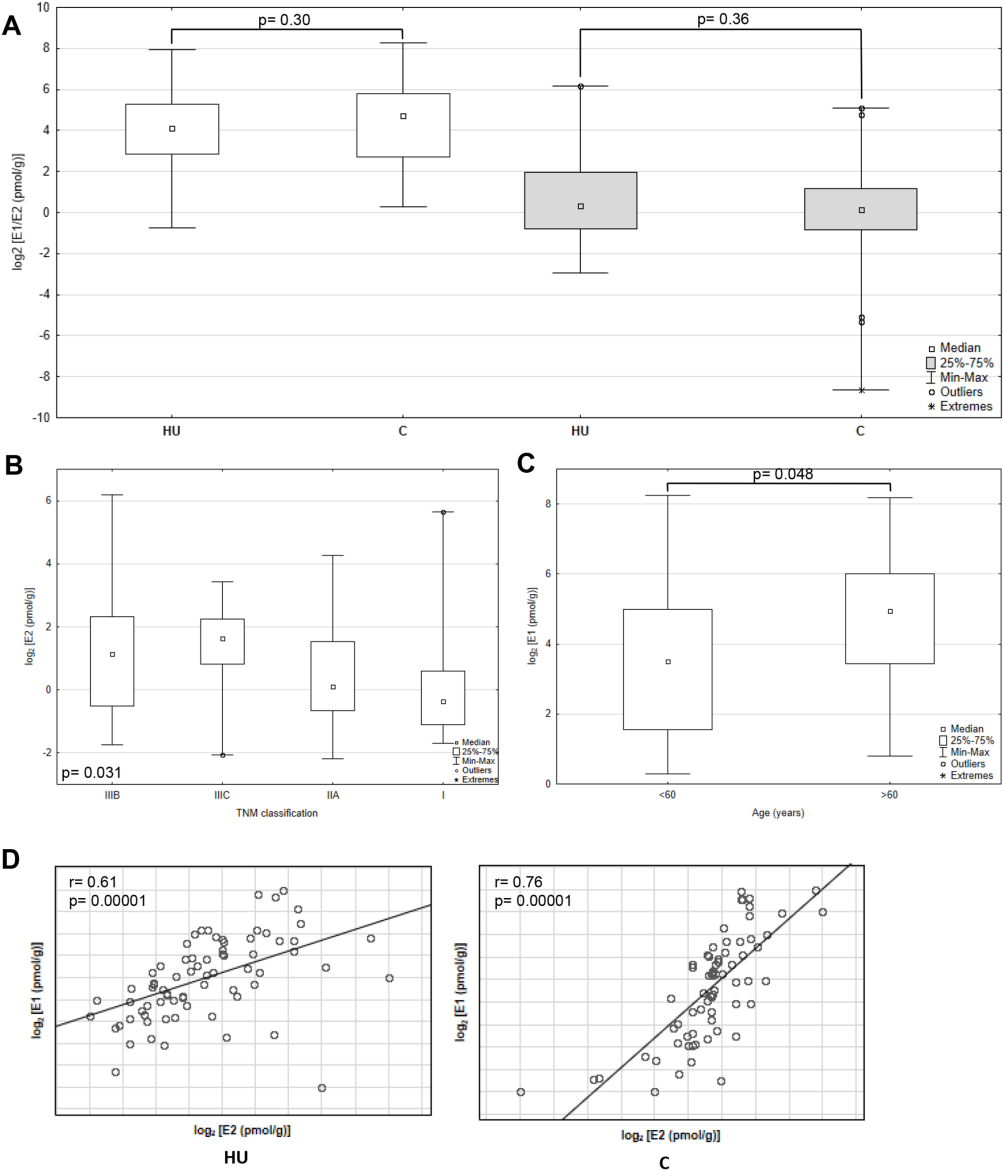
Intratissue estrone (E1) and estradiol (E2) concentrations in primary cancerous and histopathologically unchanged tissues from patients with CRC **(A)** The cancerous (C) and histopathologically unchanged tissues (HU) from 75 patients with CRC were used for steroid fraction isolation, derivatization and measurement using ESI LC/MS. E1- white boxes; E2- grey boxes. **(B)** E2 concentrations isolated from cancerous tissue classified according to TNM: group IIIB, IIIC, IIA and I. **(C)** E1 concentrations isolated from cancerous tissue classified according to patients age at the time of tumor resection. **(D)** Correlation of intratissue E1 and E2 concentrations isolated from cancerous (C) and histopathologically unchanged tissues (HU) from 75 patients with CRC. The amounts of E1 and E2 are presented as log_2_- transformed data.

The levels of intratissue estrogens were measured in 75 patients in cancerous and adjacent histopathologically unchaged tissue using ESI LC/MS. The median values for E1 were 1.5- fold higher in cancerous tissue compared with histopathologically unchanged (Figure 1A). However, observed trend have not reach statistically significant threshold probably due to high divergence of samples (range: 1.22-304.98 pmol/g for cancerous tissue; 0.59-250.18 pmol/g for histopathologcally unchanged; p=0.30). In the same sample set E2 concentration was only 1.15 higher in histopathologically unchanged tissue without statistical significance (p=0.36) with sample range 0.13-72.98 pmol/g for histopathologically unchanged and 0.0025-34.61 pmol/g for cancerous tissue. No statistical change was also identified in different age groups, genders, CRC localizations and histologic grades for E1 and E2 (Supplementary Table 1). TNM stratification revealed statistically significant higher E2 concentration in histopathologically unchanged tissue than cancerous in IIIB group (Supplementary Table 1). However, the detected outcome is an effect of other observation. Categorical clustering for histopathologically unchanged tissue indicated significant differences (p=0.031) with bias toward higher E2 concentration in IIIB and IIIC group compared with I and IIA (not significant in post-hoc analysis, Figure 1B; Supplementary Table 2). Moreover, the E1 concentration was significantly higher in patients above 60 yrs within cancerous tissue (post-hoc=0.01; Figure 1C, Supplementary Table 2).

**Figure 2:**
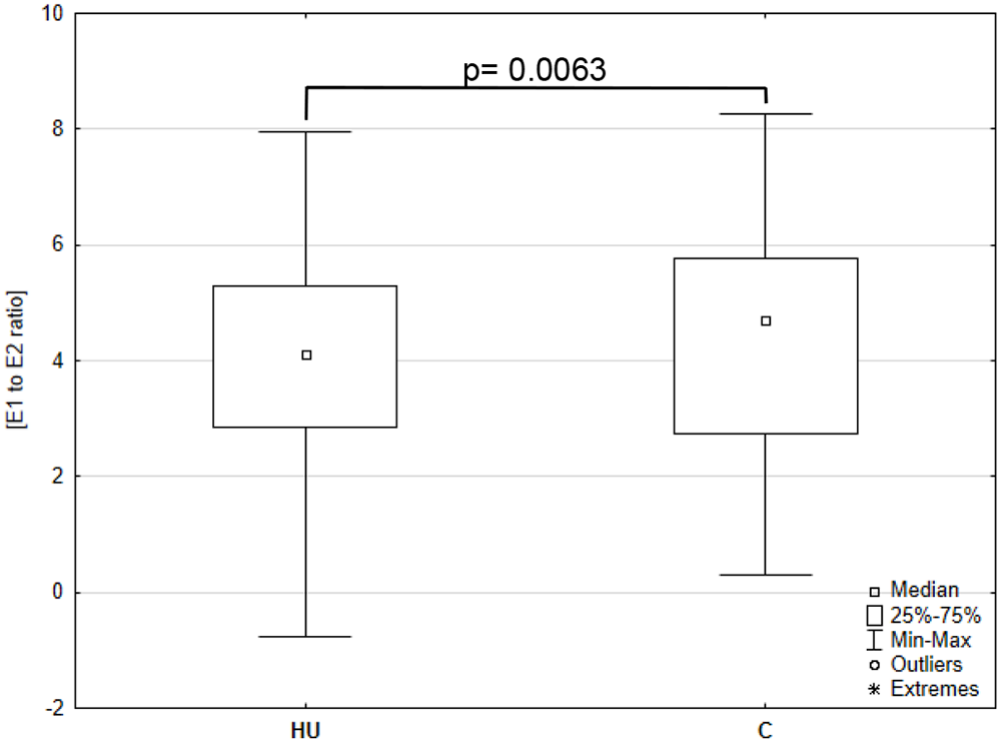
Intratissue estrone (E1) to estradiol (E2) ratio in primary cancerous and histopathologically unchanged tissues from patients with CRC The cancerous (C) and histopathologically unchanged tissues (HU) from patients with CRC were used for steroid fraction isolation, derivatization and measurement using ESI LC/MS. E1 to E2 ratio was given for the 67 samples in cancerous and 73 in histopathologically unchanged CRC tissue.

Importantly, E1 and E2 intratumoral levels were significantly correlated in histopathologically unchanged and cancerous tissue (Figure 1D). Subsequently, we analyzed E1 to E2 ratio from the same samples. We have observed that E1 to E2 ratio increased markedly in cancerous tissue compared with histopathologically unchanged (p=0.0063; Figure 2). Furthermore, we observed statistically higher E1 to E2 ratio in: patients above 60 years; men; tumor localized in proximal colon and rectum; histological grade G2 and G3 as well patients with TNM grade IIIB (Supplementary Table 3). At the same time, categorical analysis did not show statistically significant bias toward any group (Supplementary Table 2).

### ESR1 and ESR2 mRNA level is decreased in primary cancerous tissue compared with histopathologically unchanged from 110 patients with CRC

**Figure 3:**
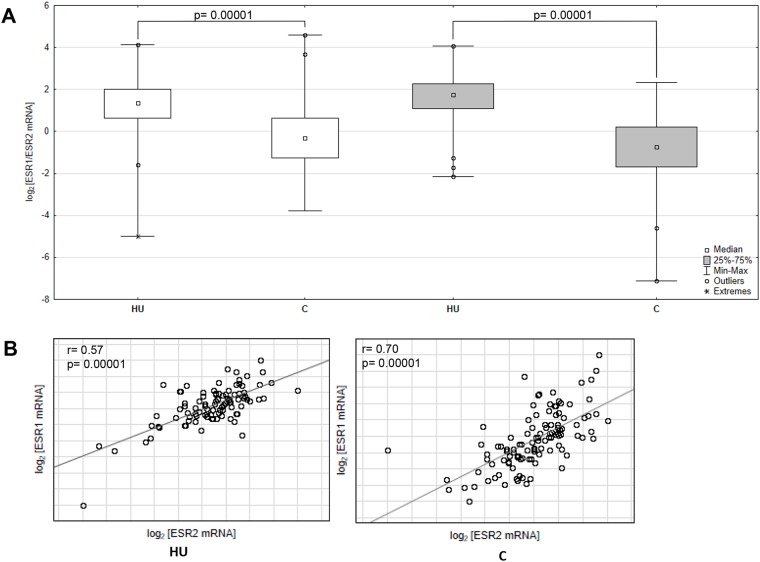
ESR1 and ESR2 transcript levels in primary cancerous and histopathologically unchanged tissue from patient with CRC **(A)** The primary cancerous tissues from 110 patients with CRC were used for RNA isolation. Total RNA was reverse-transcribed, and cDNAs were investigated by RQ-PCR relative quantification analysis. The ESR1 and ESR2mRNA levels were corrected by the geometric mean of PBGD and hMRPL19 cDNA levels. ESR1- white boxes; ESR2- grey boxes. **(B)** Correlation of ESR1 and ESR2 transcript levels from cancerous (C) and histopathologically unchanged tissues (HU) from 110 patients with CRC. The amount of ESR1 and ESR2 mRNA was expressed as the log_2_ of multiples of cDNA copies in the calibrator.

To evaluate the ESR1 and ESR2 transcript level in cancerous and histopathologically unchanged tissues from one hundred ten patients with CRC we used RT-qPCR. We found significantly lower level of ESR1 and ESR2 transcript (p< 0.0001) in primary cancerous than in the histopathologically unchanged tissues in patients with CRC (Figure 3A). Moreover, we observed significantly lower level of analyzed mRNAs in cancerous tissues in different age groups, genders, CRC localizations, histologic grades and TNM stages (Supplementary Table 4). We also detected statistically significant correlation of ESR1 and ESR2 mRNA levels in histopathologically unchanged and cancerous tissue (Figure 3B).

### Correlation between intratumoral estrogens concentrations and estrogens receptors mRNA levels with clinical outcome of CRC patients

To investigate the effect of ESR1 and ESR2 transcript levels and E1 and E2 concentrations on patients’ survival we carried out retrospective clinical analysis. The median overall survival (OS) was 49 months (range: 4-80 months) and DFS 40 (range: 5-80). Based on RT-qPCR for ESR1 and ESR2 mRNA levels and on ESI LC/MS results for E1 and E2 concentration, cancerous tissue measurements were subdivided into three groups of similar size: low, intermediate and high transcript levels or estrogen concentration. Univariate analysis revealed no benefits of high ESR1 or ESR2 mRNA levels for patients’ OS and DFS survival (Supplementary Figure 1). Moreover, even though median survival age was longer for group of patients with higher E1 and E2 concentration, the results were not statistically significant considering completed and censored patients’ cases (Supplementary Figure 1). Similarly, there was no evidence of impact of E1 to E2 ratio on OS and DFS in cancerous tissue (Supplementary Figure 1).

**Figure 4:**
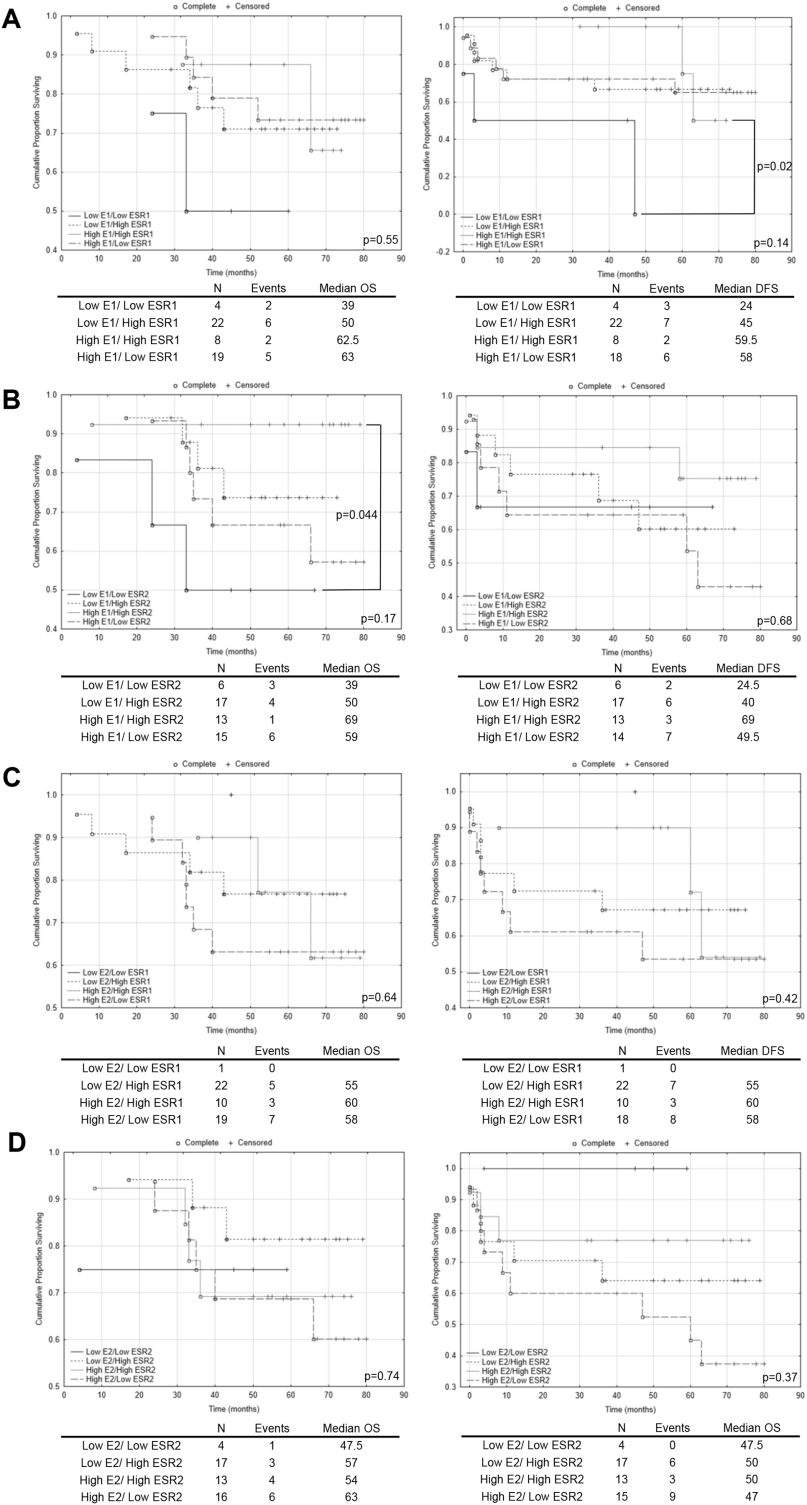
The Kaplan–Meier survival analysis among patients with colorectal cancer according to the estrogen concentration coupled with estrogen receptor transcript level Patients samples from colorectal cancerous tissue were subdivided into four categorical groups: low E1(E2) and low ESR1(ESR2); low E1(E2) and high ESR1(ESR2); high E1(E2) and high ESR1(ESR2); high E1(E2) and low ESR1(ESR2). **(A)** - comparison of categorical groups for E1/ESR1; **(B)** - comparison of categorical groups for E1/ESR2; **(C)** - comparison of categorical groups for E2/ESR1; **(D)** - comparison of categorical groups for E2/ESR2. p values for overall (OS) and disease free survival (DFS) for multiple groups comparison were given in each graph bottom right. Individual comparison of 2 groups was determined with the log rank test and given only for significant results. N, number of patients.

To better explore prognostic potential of intratissue estrogen in CRC we associated this etiological factor with molecular subgroups (expression levels of ESRs). Samples were subdivided into four categorical groups: low E1(E2) and low ESR1(ESR2); low E1(E2) and high ESR1(ESR2); high E1(E2) and high ESR1(ESR2); high E1(E2) and low ESR1(ESR2). Interestingly using log rank test, we observed that patients with coupled low E1 concentration and low ESR1 mRNA level had a significant increase in disease recurrence compared with patients with high E1 concentration and high ESR1 mRNA level (p=0.02; Figure 4A). This related to survival: 24 months in first subgroup versus 59.5 in second one (Figure 4A). Additionally, we observed benefit of high E1 concentration combined with high ESR2 transcript level for patients’ OS. There was a 20 months’ increase in OS survival compared with subgroup with low in both E1 concentration and ESR2 transcript level (Figure 4B). Although, result for E1 combined with ESR1 for OS was statistically insignificant, it suggests that there may have been a reduction in the risk of death for patients with high E1 concentration and ESR1 transcript level (Figure 4A). Combination of E1 with ESR2 for DFS and all E2 related groups disclosed lack of impact on OS and DFS survival (Figure 4C, 4D). Following, multivariate Cox regression analysis with respect to age, gender and post-operative chemotherapy status revealed that ESR1 mRNA level and E1 concentration can be together prognostic factors for patient’s survival (Table 1). We observed moderate effect of high E1 coupled with high ESR1 transcript level on patients’ OS with HR equal 0.15 (95% CI: 0.02-1.25; p=0.08) (Table 1). The effect was also significantly beneficial for any combination of high ESR1 transcript or E1 concentration for patients’ DFS (Table 1). The effect of E1 coupled with ESR2 on patients’ OS was not preserved in multivariate analysis (Table 1).

**Table 1:** Multivariate analysis of E1/ESR1 and E1/ESR2 groups in cancerous tissue in patients with colorectal cancer

Variable	OS		DFS	
	HR (95% CI)	p	HR (95% CI)	p
**E1/ESR1**				
Low E1/Low ESR1	1		1	
Low E1/High ESR1	0.29 (0.05-1.73)	0.17	0.23 (0.05-0.97)	0.04
High E1/High ESR1	0.32 (0.04-2.53)	0.28	0.16 (0.02-1.05)	0.06
High E1/Low ESR1	0.15 (0.02-1.25)	0.08	0.25 (0.06-1.16)	0.08
**Gender**				
Male	1		1	
Female	0.93 (0.22-3.99)	0.92	0.52 (0.15-1.74)	0.29
**Age**				
below 60	1		1	
above 60	1.08 (0.02-5.78)	0.93	1.92 (0.39-9.41)	0.42
**Therapy**				
no	1		1	
yes	1.31 (0.35-4.97)	0.69	1.14 (0.40-3.31)	0.79
**Variable**	**OS**			
	**HR (95% CI)**		**p**	
**E1/ESR2**				
Low E1/Low ESR2	1			
Low E1/High ESR2	0.41 (0.07-2.54)		0.34	
High E1/High ESR2	0.13 (0.01-1.91)		0.14	
High E1/Low ESR2	0.56 (0.09-3.47)		0.54	
**Gender**				
Male	1			
Female	1.22 (0.27-5.47)		0.80	
**Age**				
below 60	1			
above 60	0.84 (0.16-4.35)		0.84	
**Therapy**				
no	1			
yes	1.13 (0.30-4.35)		0.85	

OS- overall survival; DFS- disease free survival; HR- hazard ratio; CI- confidence intervals.

### ESR1 low mRNA level together with low E1 concentration is associated with higher transcript levels of β-catenin and cyclin D1 in cancerous tissue of CRC patients

**Figure 5:**
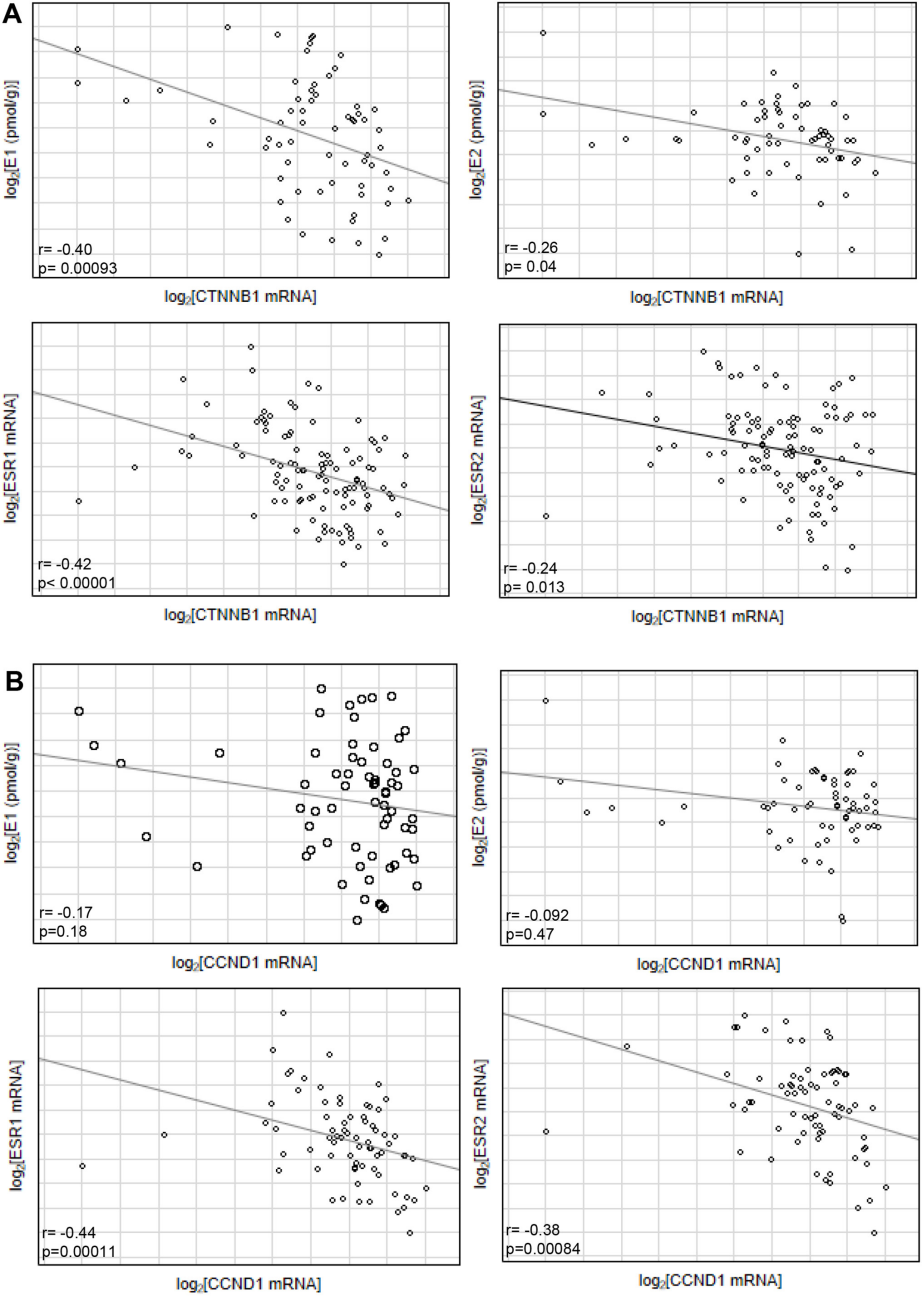
Correlation of estrogen concentration (E1/E2) or estrogen receptor transcript level (ESR1/ESR2) with CTNNB1 **(A)** and CCND1 transcript level **(B)** from cancerous tissues of CRC patients.

**Figure 6:**
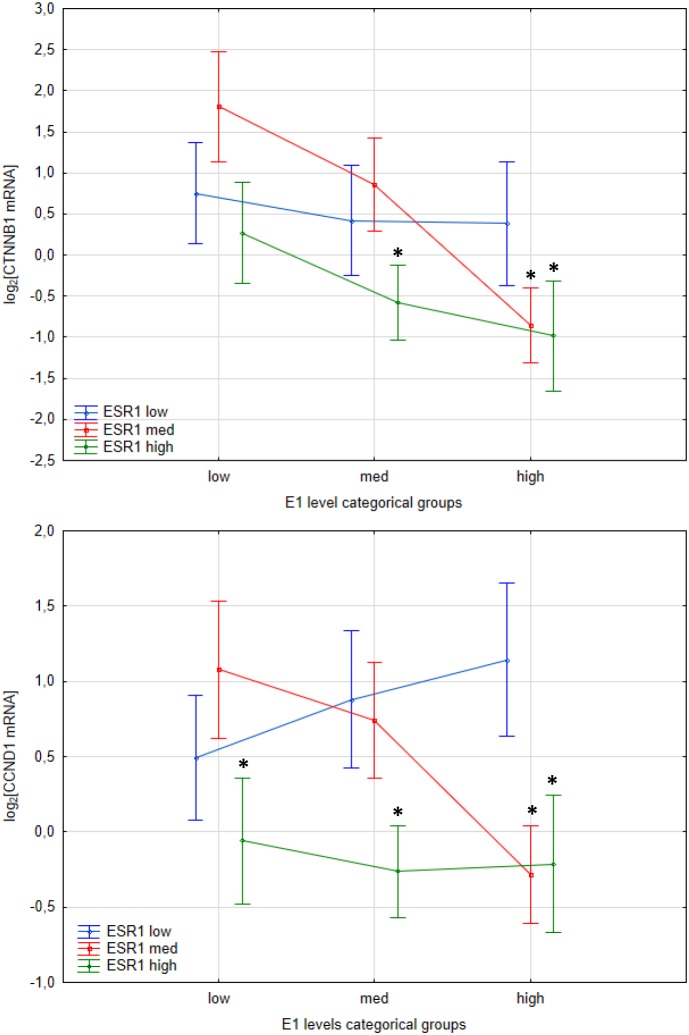
Interaction plots from two-way ANOVA presenting effect of E1 concentration level and ESR1 status on CTNNB1 and CCND1 mRNA expression level in cancerous tissues of CRC patients Values are means ± SE. Post-hoc p-values: CTNNB1: low E1-low ESR1 vs. high E1-high ESR1 p=0.06; low E1-med ESR1 vs. high E1-high ESR1 p=0.005; low E1-med ESR1 vs med E1-high ESR1 p=0.005; low E1-med ESR1 vs high E1-med ESR1 p=0.001. CCND1: high E1-low ESR1 vs low E1-high ESR1 p=0.073; high E1-low ESR1 vs med E1-high ESR1 p=0.022; high E1-low ESR1 vs high E1-high ESR1 p=0.05; low E1-med ESR1 vs high E1-med ESR1 p=0.022.

Previous studies suggest influence of estrogenic signaling on Wnt pathway. In Apc^Min/+^ mice β-catenin expression, encoded by *CTNNB1*, was higher in ER-α deficient mice, that followed overexpression of Wnt activated genes including cyclin D1, encoded by *CCND1* [11]. Hence we have evaluated effect of intratissue estrogen concentration and *ESR1* and *ESR2* expression on *CTNNB1* and *CCND1* mRNA level. As expected, expression of both *CCND1* (p=0.000016) and *CTNNB1* (p<0.000001) was strongly upregulated in CRC patients compared with histopathologically unchanged tissue (Supplementary Figure 2). Following, estrogen receptors mRNA level and estrogen concentration was inversely correlated with *CTNNB1* transcript levels whereas only estrogen receptors were negatively correlated with *CCND1* transcript (Figure 5). Moreover, CTNNB1 mRNA level was significantly reduced in cancerous tissue with simultaneously high ESR1 transcript and E1 concentration as compared with expression level in group with low E1 level and low or intermediate ESR1 mRNA level (Figure 6). For group intermediately expressing ESR1 we observed significant decrease of CTNNB1 transcript as E1 level was higher. Same inverse correlation we observed for CCND1 mRNA level (Figure 6). CCND1 transcript was significantly lower in samples expressing high ESR1 level regardless of E1 level as compared with samples with low ESR1 level and high E1 concertation (Figure 6). Significant differences were also seen for CTNNB1 transcript when we analyzed E1 together with ESR2 status (Supplementary Figure 3). We have also detected moderate inverse relation of CTNNB1 and CCND1 mRNA level with ESR1 and E2 level (Supplementary Figure 3). Surprisingly we have not observed clear influence of ESR2-E2 on CTNNB1 nor CCND1 (Supplementary Figure 3). Only subgroup intermediately expressing E2 and ESR2 presented significantly lower mRNA level of both transcripts (Supplementary Figure 3).

## DISCUSSION

Estrogen related pathways are implicated in gene expression regulation and homeostasis of many tissues and consequently are often altered in numerous pathophysiological conditions including cancer. Numerous evidences associate estrogen with CRC occurrence. Recent metaanalysis summarizes observational studies and confirm link of HRT usage with decrease in CRC incidence [14]. Even though results seems to be reproducible some reports present contrary data, which may be consequence of various observational studies aspects: usage of different hormones formulations or variation in HRT-start point in relation to menopause [15]. At the same time, studies related to blood circulating estrogens rather relate high estrogens concentrations with the CRC risk or report no relevant connection [16–19]. Only recent publication inversely associates endogenous E1 and E2 with CRC risk [20]. Estrogens are also thought to lower severity of the inflammatory bowel disease (IBD) [21, 22]. Importantly, extragonadal tissues, including colon, may produce E1 and E2 from circulating precursors by aromatase or sulfatase pathway [9]. Our and other groups’ data prove alterations of enzymes activity involved in estrogen metabolism in CRC [23–26]. Active intratissue estrogen metabolism raises the question about significance of local E1 and E2 concentrations. In this study we have not indicated statistically significant differences in analyzed estrogens concentrations between cancerous and adjacent histopathologically unchanged tissue. Even though E1 and E2 levels were correlated, ratio of E1 to E2 was significantly higher in cancerous tissue compared with histopathologically unchanged. The achieved data resemble only one similar report where 53 CRC patients’ samples were analyzed [27]. Sato et al. observed statistically significant 2-fold higher E1 concentration in cancerous tissue [27]. Our data follow this trend with statistically insignificant 1.5- higher E1 concentration in cancerous tissue compared with histopathologically unchanged. However, we have observed statistically significant higher E1 concentration within cancerous tissue for patients above 60 yrs old, regardless of their gender. In context of intratissue estrogen metabolism, higher E1 concentration and alterations in E1 to E2 ratio in cancerous tissue may follow our previous observations. Expression of *17β- hydroxydehydrogenase type 1* (*HSD17β1*)- gene encoding main enzyme responsible for converting E1 into E2 was reduced in CRC tissue [24]. However, English et al. found in CRC loss of *HSD17β2* expression responsible for oxidation E2 to E1 [26]. Our data suggest also decrease of E1-S desulfonation enzyme [28]. The conflicting data from previous reports as well observed in this study high divergence of estrogen concentrations among tissues suggests that the intratissue E1 and E2 level may be affected by etiological factors like obesity. Adipose tissue contributes meaningfully to extragonadal steroid hormones production and may regulate amount of precursors available for estrogen metabolism in colon tissues [29]. Moreover, actual Km values of enzymes involved in estrogen metabolism, not only their amount, should be investigated in future more deeply in CRC tissue. Furthermore, in contradiction to the Sato report we have not observed any effect of the E1 or E2 concentration on CRC patients’ clinical outcome [27]. The discrepancies might be an effect of the different group size or patient’s medical history. Our study enrolled only women that were non- contraceptive users and have not taken HRT, which was not pointed out in the referred publication.

Estrogen action is mediated by its specific receptors which implication in CRC has been also widely investigated. ER type α and β are nuclear receptors that after dimerization and ligand binding translocate to nucleus where initiate transcription of genes containing estrogen response element (ERE) in the promoter region [30]. Moreover, ERs can cross-talk with other transcription factors complexes and affect non-ERE containing genes. In colon mainly expressed form is ER-β with limited expression of ER-α [12]. Overwhelming amount of evidence, summarized in recent review, prove inverse relationship of ER-β presence with occurrence of CRC or familial adenomatous polyposis [13]. Immunohistochemistry based studies of large group of CRC patients’ associate low ER-β expression with poorer OS and DFS survival [31–33]. Additionally, studies on IBD presented lower *ESR2* expression levels in colonic mucosa of Crohn’s disease (CD) and ulcerative colitis (UC) patients compare with controls [34, 35]. Reduced *ESR1* expression caused by DNA hypermethylation in promoter region was also observed in UC patients with neoplastic regions [36]. In line with previous reports, transcript levels of *ESR1* and *ESR2* were reduced in our study in cancerous tissue compared with histopathologically unchanged. However, ESR1 and ESR2 transcript levels presented lack of impact on patient survival. To best of our knowledge, this is first report investigating ESR1 next to ESR2 mRNA level in context of CRC patients’ survival.

Even though *ESR2* seems to be main isoform expressed in colon we have observed that mRNA levels of both: ESR1 and ESR2 were reduced and correlated which suggest that decrease of both may occur equivalently during CRC progression. To date, *in vitro* studies focused on *ESR2* expression effect on CRC cells proliferation and expression of oncogenes or tumor suppressors, revealing it protective role [13]. For example, ER-β has been shown to induce apoptosis in CRC cell lines through upregulation of p53 signalling and regulation G1- specific cell cycle genes [37, 38]. *In vitro* data regarding *ESR1* expression are limited. One report suggests that overexpression of *ESR1* in CRC cell lines have antiproliferative function [39]. More data regarding function of both receptors in CRC is available from *in vivo* studies. Apc^Min/+^ mice knockouts for ER-α or -β had higher tumour formation than respective controls in independent experiments [10, 11, 40, 41]. Correspondingly, treatment Apc^Min/+^ mice with ER- selective agonists resulted in repression of CRC development [42, 43].

To identify actual estrogen responsive CRC tissue and predict impact on patient clinical outcome we performed categorical clustering of E1 and E2 concentration with ERs transcript levels. Interestingly, log rank test revealed beneficial DFS effect of coupled high E1 intratissue concentration with high ESR1 mRNA level compared with respective low E1 and ESR1 group. Additionally, multivariable analysis suggests that E1 concentration coupled with ESR1 transcript status might be independent prognostic factor. Even though results have to be validated in large studies with extended follow up, the findings are biologically plausible and for the first time link local estrogen concentration with ER status. Although E1 is not main biologically active form of estrogen, it may bind to ERs [44]. Moreover, E1 was shown to have antiproliferative effects in CRC cells [26] and decrease carcinogen induced- CRC incidence in ovariectomized mice [7]. Surprisingly no statistically significant correlation was found for category of E2 together with any of ER isoform. Observed result could be an effect of limited number of patients in given groups. Subgroup: low E2/low ESR1 had only one patient case and group: low E2/low ESR2 contained mostly censored data. Based on that we could not have evaluated this intriguing issue. To date, only one study presented significant lower CRC risk for patient with HRT history use coupled with ESR2-positive immunohistochemistry staining [45].

To verify protective effect of E1 coupled with ESR1 we have evaluated estrogen dependent mechanisms involved in CRC. Aberrant activation of Wnt signalling pathway is frequently observed in CRC [46] and former studies suggested influence of estrogenic pathway on Wnt in CRC. *β-catenin* expression in Apc^Min/+^ mice was higher in ER-α deficient mice, but not ER-β, that followed upregulation of Wnt activated genes including cyclin D1 and c-myc [11, 47]. Supporting observations from *in vivo* studies we have detected the highest transcript level of cyclin D1 and β-catenin in CRC patients with low or intermediate ESR1 transcript level together with low E1 concentration. The similar trend was also observed for β-catenin transcript level with E1-ESR2 subgroups but not for E2-ESR2 classification for both genes. Expression of *β-catenin* and *cyclin D1* might be upregulated in CRC in independently of estrogenic pathway, nevertheless the inverse relation of their expression to E1-ESR1 status follow previous observation on animal models. Hence, it seems that in opposition to Wnt-estrogen regulation in breast cancer [48], ER-α has different effects in colon. Still, exact molecular mechanisms of ligand dependent ER-α action in CRC are not deeply described.

Present study for the first time provides evidence for association between intratissue estrogen concentrations coupled with estrogen receptor status. Importantly, it indicates potential prognostic values for etiological factor combined with molecular changes. Unfortunately, due to small amount of tissue sample we could have not assess ER protein levels. Limitation of our analysis could be also lack of more detailed data on other factors, both etiological and molecular, that may influence local estrogen synthesis. Future studies in large CRC patient cohort should stratify for other factors as well measure intratissue activity of enzymes involved in steroidogenesis in CRC tissue.

In conclusion, our data associate high ESR1 mRNA level together with high E1 intratissue concentration with better DFS of CRC patients. The protective effects of E1-ESR1 might be switched off in subgroup of CRC patients, indicated by higher expression of *β-catenin* and *cyclin D1*, possibly explaining correlation of high ESR1-E1 level with better DFS. These results are in line with hypotheses presented mostly in animal studies and emphasize significance of tissue estrogen concentration in relation to ERs expression. Moreover, data suggest potential usage of these factors for clinics, either as biomarkers or as targets for estrogen related therapeutics.

## MATERIALS AND METHODS

### Patient material

Primary colonic adenocarcinoma tissues were collected between June 2009 and March 2013 from one hundred ten patients who underwent radical surgical resection of the colon at the Department of General and Colorectal Surgery, Poznań University of Medical Sciences, Poland (Supplementary Table 5). The histopathologically unchanged colonic mucosa located at least 10-20 cm away from the cancerous lesions was obtained from the same patients. One set of samples was immediately snap-frozen in liquid nitrogen and stored at −80°C until estrogen/RNA isolation. The other set of samples was directed for histopathological examination. Histopathological classification was performed by an experienced pathologist. None of analyzed patients received preoperative chemo- or radiotherapy. All females were in postmenopausal age and none was using oral contraceptives or received hormone replacement therapy. An informed consent was obtained from all participating individuals. The procedures of the study were approved by the Local Ethical Committee of Poznań University of Medical Sciences.

### Measurement of overall and disease free survival

Follow-up data were available for eighty two patients, who were observed from 2009/08/11 until death or 2016/02/10, whichever came first. Disease free survival (DFS) is defined as the time elapsed from surgery to the first occurrence of any of the following events: recurrence or distant metastasis of CRC or development of a second non-colorectal malignancy.

### Liquid chromatography electrospray ionization tandem mass spectrometry analysis (ESI LC/MS)

Tissue specimens (up to 40 mg per sample) were homogenized in Freezer/Mill, SPEX SamplePrep (Metuchen NY, USA) and dissolved in 1 ml of distilled water. At this step C13 derivatives of E1 and E2 were added to each sample as internal standards. Steroid fraction was extracted from tissue using diethyl ether. Obtained organic layer was evaporated and subsequently derivatized with dansyl chloride [49, 50].

All samples were analyzed using an LC/MS system built on Waters Nano Acquity UPLC, Waters (Milford MA, US) combined with a Ion Trap mass spectrometer, model Amazon SL, Bruker Daltonics (Bremen, Germany). Analyses were carried out using nanoAcquity Symmetry C18 column, Waters (3.5μm, 150μm x 150mm). Chromatographic separation was performed at a flow rate of 8 μL/min using mixtures of the following two solvents: A (99.5% H2O, 0.5% formic acid (v/v)) and B (99.5% acetonitrile, 0.5% formic acid (v/v)). The column effluent was introduced into an ESI ion source using the gradient as follows: 0–5 min isocratic separation at 5% B, 5-15 min gradient from 5 to 45% B, 15–20 min linear gradient to 95% B, 20–25 min of isocratic flow at 95% B and for 5 additional min return to the initial conditions.

The Amazon SL spectrometer consisted of ESI operating at - 4.5 kV, nebulization with nitrogen at 1.6 bar and a dry gas flow of 8.0 L/min at a temperature of 220°C. The analyser operated at MRM mode, 4 different transitions were measured simultaneously: 504->171 and 506->171 for E1; 507->171 and 509->171 for E2 (Supplementary Figure 4). The instrument was scanning in Enhanced Resolution mode under the control of trapControl version 7.1, and data were analyzed using the DataAnalysis version 4.1 package supplied by Bruker Daltonics. Profiles of E1, E2 and their deuterated forms were extracted for specific transitions and areas under peaks were measured. Quantitative analysis was performed by preparation of calibration curve for E1 and E2 in concentration range from 10 fg/ml to 10 ng/ml. The curve showed high linearity in given range (R^2 > 0.95) allowing confident measurement of E1 and E2 concentrations in the tissue samples.

### Reverse transcription and real-time quantitative polymerase chain reaction (RQ-PCR) analysis

Total RNA from tissues of patients with CRC was isolated according to the method of Chomczyński and Sacchi [51]. The 1 μg of each RNA sample was reverse-transcribed into cDNA using SuperScript™ reverse trascriptase, ThermoFisher Scientific (Waltham, USA). RT-qPCR was carried out in the Light Cycler®480 Real-Time PCR System, Roche Diagnostics GmbH (Mannheim, Germany) using EvaGreen as the detection dye. The target cDNA was quantified by the relative quantification method using a calibrator. The calibrator was prepared as a cDNA mix from all of the patients’ samples, and successive dilutions were used to create a standard curve as described in Relative Quantification Manual Roche Diagnostics GmbH, (Mannheim, Germany). For amplification, 1 μl of (total 20 μl) cDNA solution was added to 9 μl of 5 X Hot FIREPol EvaGreen HRM Mix, Solis BioDyne Co. (Tartu, Estonia) with primers (Supplementary Table 6). To prevent amplification of sequences from genomic DNA contamination, primers and/or amplicons were designed at exon/exon boundaries and covered all gene splice variants. The quantity of ESR1, ESR2, CTNB1 and CCND1 transcripts in each sample was standardized by the geometric mean of two internal controls: *porphobilinogen deaminase* (*PBGD*) and *human mitochondrial ribosomal protein L19* (*hMRPL19*) (Supplementary Table 6). The selection of internal control genes was made as previously [23]. The ESR1, ESR2, CTNB1 and CCND1 transcript level in the patients’ tissues were expressed as multiplicity of the cDNA concentrations in the calibrator.

### Statistical analysis

The normality of the observed patient data distribution was assessed by Shapiro-Wilk test, and the U Mann-Whitney test was used to compare the median values. Non-parametric Kruskal-Wallis test with post-hoc multiple comparison of mean ranks was employed to evaluate the association between different categorical groups within cancerous or histopathologically unchanged tissue for estrogen concentrations and ESR1/ESR2 mRNA levels. Correlation between the analyzed data was performed using Spearman rank correlation coefficient. Additionally, two-way ANOVA following post-hoc test was performed to assess the interaction of estrogen-estrogen receptors and CTNNB1 or CCND1 transcript level.

Survival curves were plotted using the Kaplan-Meier method and survival differences were achieved using the log-rank test. Multivariate Cox proportional hazard model was used to estimate the adjusted hazard ratio (HR).

Statistically significant results were indicated by p < 0.05. Statistical analysis was performed with STATISTICA 10.0 software.

## SUPPLEMENTARY FIGURES AND TABLES









## Abbreviations

CCND1: cyclin D1
CTNBB1: β-catenin
CRC: colorectal cancer
DFS: disease free survival
E1: estrone
E1-S: estrone sulfate
E2: 17β- estradiol
ER (gene ESR1 ESR2): estrogen receptor
ESI LC/MS: Liquid chromatography electrospray ionization tandem mass spectrometry
hMRPL19: human mitochondrial ribosomal protein L19
HR: hazard ratio
HRT: hormone replacement therapy
HSD17βs: 17β-hydroxysteroid dehydrogenases
OS: overall survival
PBGD: porphobilinogen deaminase
RQ-PCR: real-time quantitative polymerase chain reaction
