# Supplementary material for: Significance of intratissue estrogen concentration coupled with estrogen receptors levels in colorectal cancer prognosis
Source: Oncotarget. 2017 Dec 14;8(70):115546–60. doi: 10.18632/oncotarget.23309 (PMC5777792; doi:10.18632/oncotarget.23309)
Supplement: Supplementary file 1 [file oncotarget-08-115546-s001.pdf]

Significance of intratissue estrogen concentration coupled with estrogen receptors levels in colorectal cancer prognosis

SUPPLEMENTARY MATERIALS

A

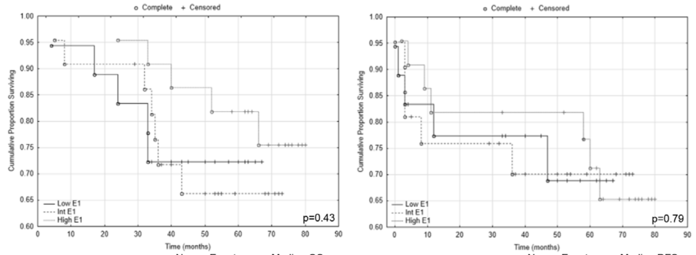

	N	Events	Median OS
Low E1	18	5	47.5
Int E1	22	7	51.5
High E1	22	5	69

	N	Events	Median DFS
Low E1	18	5	46
Int E1	21	6	40
High E1	22	7	63.5

B

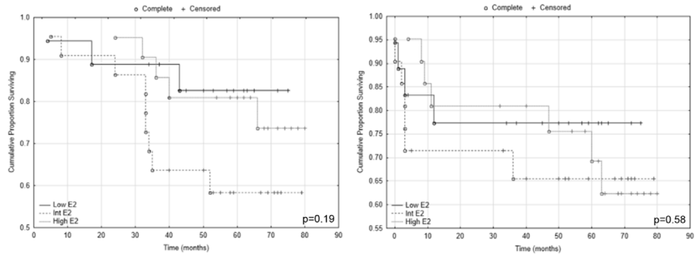

	N	Events	Median OS
Low E2	18	3	51.5
Int E2	22	9	52.5
High E2	21	5	66

	N	Events	Median DFS
Low E2	18	4	50
Int E2	21	7	40
High E2	21	7	62

C

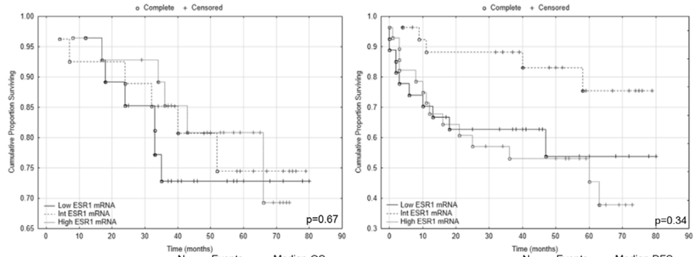

	N	Events	Median OS
Low ESR1 mRNA	28	7	39
Int ESR1 mRNA	27	6	50
High ESR1 mRNA	28	6	51.5

	N	Events	Median DFS
Low ESR1 mRNA	27	11	33
Int ESR1 mRNA	27	5	50
High ESR1 mRNA	28	15	35

D

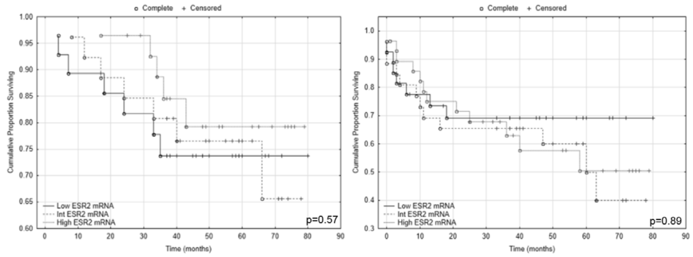

	N	Events	Median OS
Low ESR2 mRNA	28	7	40
Int ESR2 mRNA	26	7	52.5
High ESR2 mRNA	28	5	45.5

	N	Events	Median DFS
Low ESR2 mRNA	27	8	37
Int ESR2 mRNA	26	12	40.5
High ESR2 mRNA	28	12	35

(Continued)

E

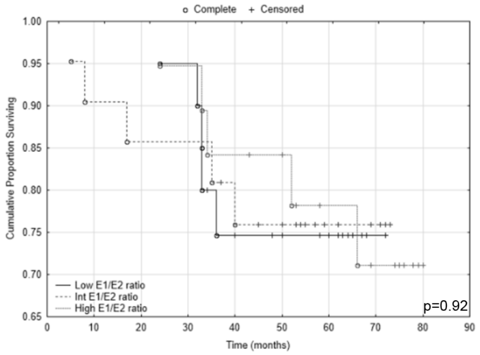

	N	Events	Median OS
Low E1/E2 ratio	20	5	54
Int E1/E2 ratio	21	5	55
High E1/E2 ratio	19	5	69

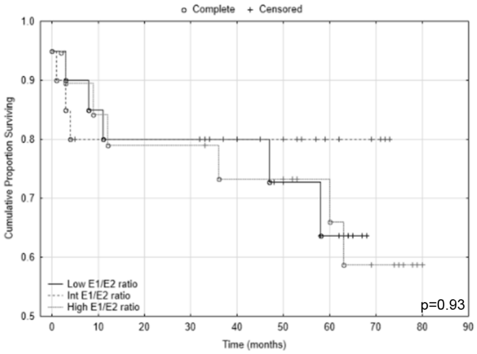

	N	Events	Median DFS
Low E1/E2 ratio	20	6	47.5
Int E1/E2 ratio	20	4	55.5
High E1/E2 ratio	19	7	60

Supplementary Figure 1: The Kaplan–Meier survival analysis among patients with colorectal cancer according to the estrogen concentration **(A** and **B)**, estrogen receptor transcript level **(C** and **D)** and E1 to E2 ratio **(E)**.

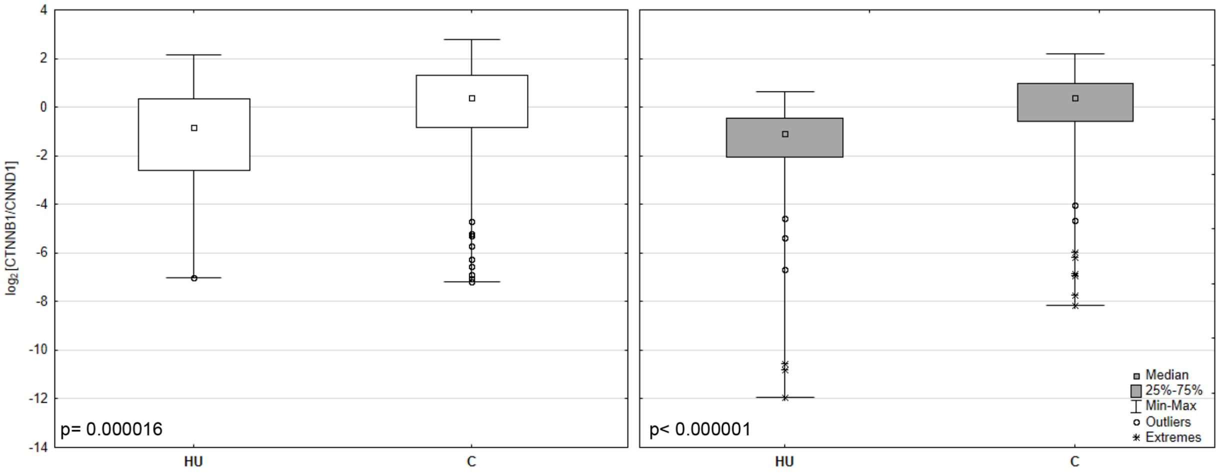

Supplementary Figure 2: CTNNB1 (white boxes) and CCND1 (grey boxes) transcript levels in primary cancerous and histopathologically unchanged tissue from patient with CRC. The primary cancerous tissues from patients with CRC were used for RNA isolation. Total RNA was reverse-transcribed, and cDNAs were investigated by RQ-PCR relative quantification analysis. The CTNNB1 and CCND1 mRNA levels were corrected by the geometric mean of PBGD and hMRPL19 cDNA levels. The amount of mRNA was expressed as the $\log_2$ of multiples of cDNA copies in the calibrator.

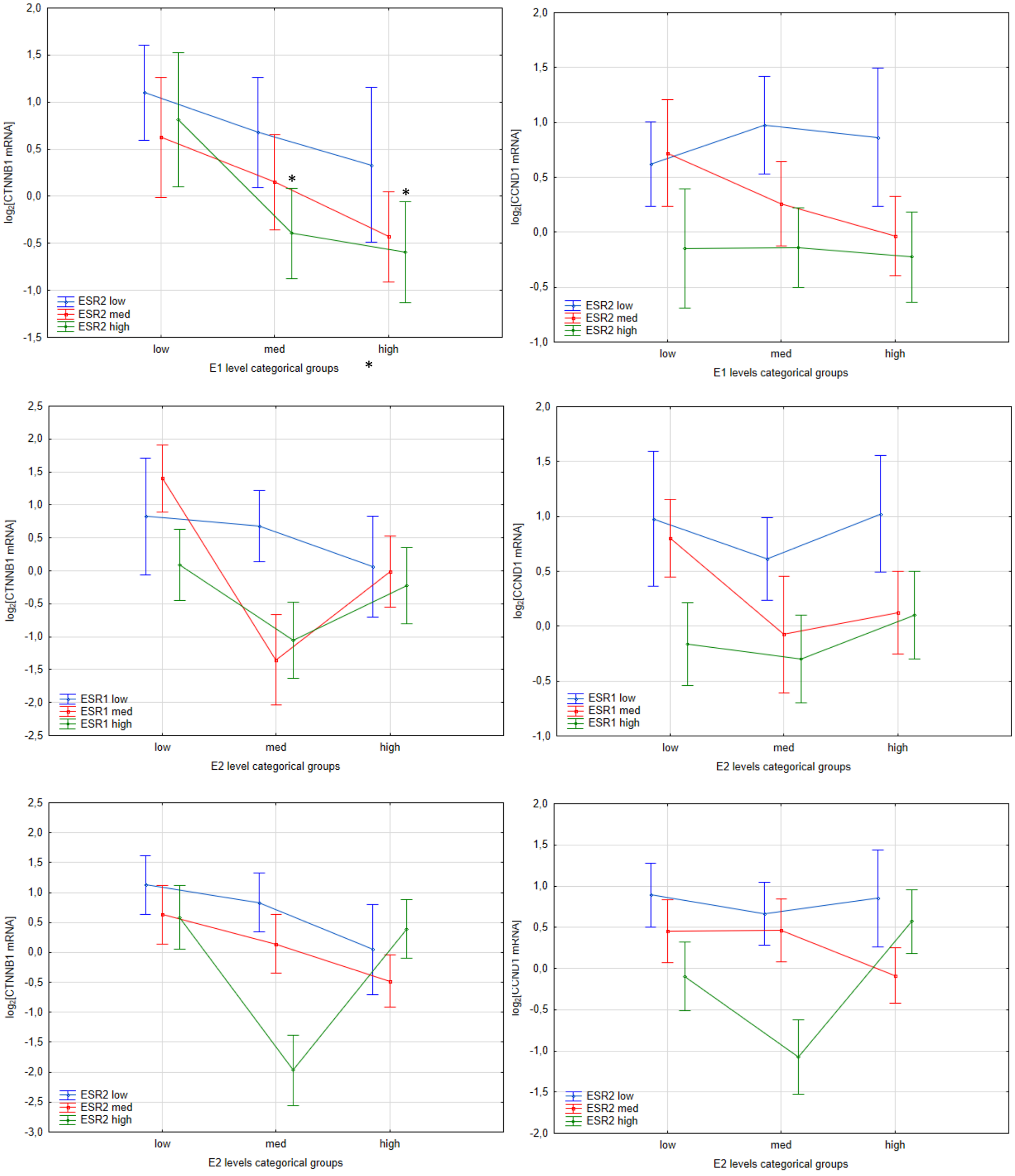

Supplementary Figure 3: Interaction plots from two-way ANOVA presenting effect of estrogen (E1 or E2) concentration level and estrogen receptor status (ESR1 or ESR2) on CTNNB1 and CCND1 mRNA expression level. Values are means ± SE.Post-hoc p-values: CTNNB1: low E1-low ESR2 vs.med E1-high ESR2 p=0.032; low E1-low ESR2 vs.high E1-high ESR2 p=0.027.

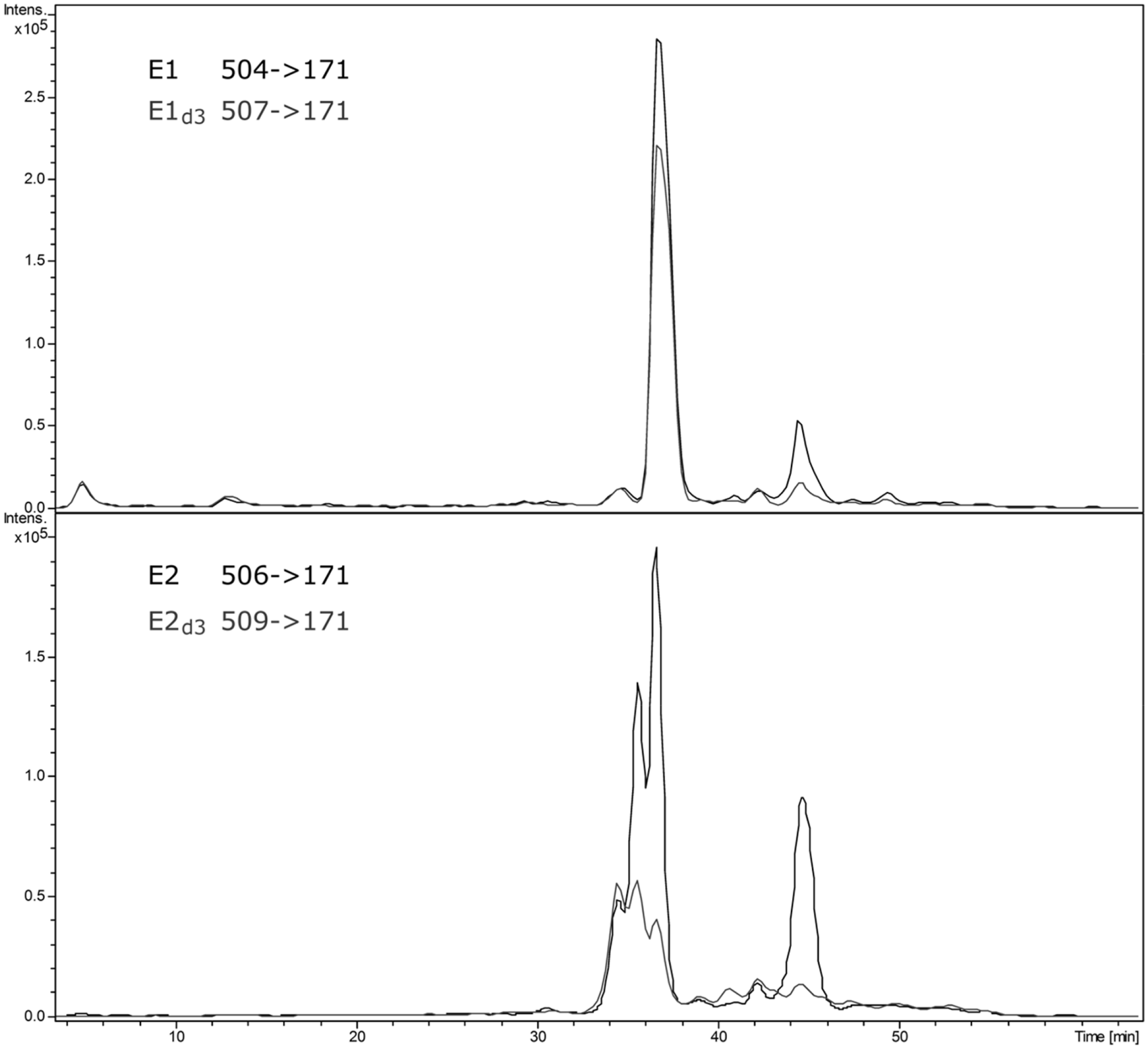

Supplementary Figure 4: MRM chromatograms of LC/MS data for E1 and E2 from cancerous tissue and C13 standards.

Supplementary Table 1: E1 and E2 concentrations in primary cancerous and histopathologically unchanged tissue samples from patients with CRC

See Supplementary File 1

Supplementary Table 2: E1 and E2 concentrations, E1 to E2 ratio and ESR1 and ESR2 transcript levels in different categorical groups within cancerous or histopathologically unchanged tissue of patient with CRC

See Supplementary File 2

Supplementary Table 3: E1 to E2 ratio in primary cancerous and histopathologically unchanged tissue samples from patients with CRC

E1 to E2 ratio	Primary cancerous tissue	Histopathologically unchanged tissue	p[a]
	median (range)		
	20.21 (1.19-489.21)	15.32 (0.033-56.69)	0.0063
**Age (years)**			
<60	13.99 (1.19-489.21)	9.83 (0.033-31.46)	0.17
>60	22.61 (2.54-110.53)	16.05 (0.24-56.69)	0.0058
**Gender**			
Female	22.46 (4.55-489.21)	15.12 (0.24-49.17)	0.076
Male	19.12 (1.19-110.53)	15.32 (0.033-56.69)	0.022
**Localization**			
Proximal colon	17.83 (3.91-489.21)	14.85 (0.033-56.69)	0.084
Distal colon	30.08 (1.19-92.15)	16.06 (1.15-54.65)	0.213
Rectum	22.05 (2.54-72.05)	15.07 (0.23-47.07)	0.017
**Histologic grade**			
G1	19.12 (11.09-52.58)	10.89 (1.16-42.38)	0.38
G2	22.32 (1.19-489.21)	16.13 (0.033-54.65)	0.027
G3	14.68 (3.91-72.05)	10.13 (0.23-47.07)	0.068
**TNM classification**			
I	15.48 (5.82-82.89)	9.66 (1.16-40.36)	0.23
IIA	17.15 (2.54-52.58)	12.3 (0.033-49.13)	0.18
IIC	36.98 (3.91-70.05)	7.32 (4.30-10.34)	-
IIIB	28.19 (4.93-489.21)	19.90 (0.23-47.07)	0.027
IIIC	8.46 (1.19-22.44)	9.99 (1.15-16.04)	0.78

Supplementary Table 4: ESR1 and ESR2 transcript levels in primary cancerous and histopathologically unchanged tissue samples from patients with CRC

See Supplementary File 3

Supplementary Table 5: Clinicopathological characteristics of patients with colorectal cancer

Features	No. of patients
**Total no. of patients**	110
**Gender (Female/Male)**	47/63
**CRC localization**	
Proximal colon (cecum to transverse)	39
Distal colon (splenic flexure to sigmoid)	21
Rectum	50
**Histological grade**	
G1	6
G2	68
G3	36
**TNM classification**	
I	17
IIA	40
IIC	2
IIIA	3
IIIB	34
IIIC	14

Supplementary Table 6: Primer sequences

Primers	Sequence (5'-3')	UCSC position (GRCh37/hg19)	Product size (bp)
	**Primers for RQ-PCR**		
ESR1	F: GGAGTGTACACATTTCTGTC<br>R: CAAAGTGTCTGTGATCTTGTC	chr6:152 415 519-152 415 608	90
ESR2	F: GTTCTGGACAGGGATGAG<br>R: CCTTGACACAGAGATATTC	chr14:64 716 285-64 723 954	124
CTNNB1	F: TGGCTACTCAAGCTGATTTGA<br>R: TGGTAGTGGCACCAGAATGG	chr3:41 265 561-41 266 127	123
CCND1	F: GAAGGAGACCATCCCCCTGA<br>R: GAAATCGTGCGGGGTCATTG	chr11:69 457 939-69 458 665	142
PBDG	F: GCCAAGGACCAGGACATC<br>R: TCAGGTACAGTTGCCCATC	chr11:118 468 348-118 468 864	160
MRPL19	F: ACTTTATAATCCTCGGGTC<br>R: ACTTTCAGCTCATTAACAG	chr2:75 735 389- 75 735 705	171

F- forward; R-reverse.
