# Supplementary material for: Significance of intratissue estrogen concentration coupled with estrogen receptors levels in colorectal cancer prognosis
Source: Oncotarget. 2017 Dec 14;8(70):115546–60. doi: 10.18632/oncotarget.23309 (PMC5777792; doi:10.18632/oncotarget.23309)
Supplement: Supplementary file 2 [file oncotarget-08-115546-s002.doc]

| **E1 (pmol/g)** | **Primary cancerous tissue** | **Histopathologically unchanged tissue** | **p^a^** |
| --- | --- | --- | --- |
|  | median (range) | |  |
| **Age (years)**  <60 >60  **Gender**  Female  Male  **Localization**  Proximal colon  Distal colon  Rectum  **Histologic grade**  G1  G2  G3  **TNM classification**  I  IIA IIC IIIA IIIB IIIC | 26.04 (1.22-304.98)  11.34 (1.22-304.98) 30.73 (1.75-289.69)  26.05 (1.22-231.70)  27.89 (1.66-304.98)  16.83 (1.22-289.69)  30.11 (1.66-304.98) 31.19 (2.94-246.52)  24.82 (10.8-49.86)  30.94 (1.22-289.69) 15.57 (1.75-304.98)  21.67 (4.48-231.70)  28.29 (2.77-304.98) 7.67 (1.74-13.60) 51.28 23.73 (1.22-246.52) 7.88 (1.66-80.38) | 17.20 (0.59-250.18)  9.22 (0.59-59.39) 19.28 (0.96-250.18)  17.3 (2.15-72.09)  14.10 (0.59-250.18)  9.49 (0.59-250.18)  14.70 (2.71-141.4) 24.5 (2.15-214.65)  16.32 (4.78-57.69)  19.28 (0.59-250.18) 15.65 (0.96-214.65)  12.52 (2.66-57.69)  12.75 (0.59-89.88) 2.72 (0.96-4.49) 57.84 21.74 (2.20-214.64) 20.19 (2.71-141.40) | 0.30  0.90 0.10  0.47  0.26  0.12  0.42 0.61  0.59  0.30 0.78  0.27  0.13 - - 0.61 0.52 |

| **E2 (pmol/g)** | **Primary cancerous tissue** | **Histopathologically unchanged tissue** | **p^a^** |
| --- | --- | --- | --- |
|  | median (range) | |  |
| **Age (years)**  <60 >60  **Gender**  Female  Male  **Localization**  Proximal colon  Distal colon  Rectum  **Histologic grade**  G1  G2  G3  **TNM classification**  I  IIA IIC IIIB IIIC | 1.09 (0.0025-34.61)    0.85 (0.0025-27.07)  1.12 (0.025-34.61)  1.11 (0.0025-9.52) 1.09 (0.02-34.61)  1.06 (0.0025-34.61)  1.24 (0.03-27.07) 1.18 (0.13-6.04)  0.95 (0.56-2.24)  1.17 (0.0025-34.61) 1.07 (0.025-27.07)  0.98 (0.62-6.04)  1.19 (0.31-27.07) 1.75 (0.024-3.48) 1.06 (0.002-34.61) 1.21 (0.35-3.58) | 1.26 (0.13-72.98)    1.89 (0.40-17.68)  1.23 (0.13-72.98)  1.20 (0.22-72.98) 1.38 (0.13-49.74)  0.97 (0.13-49.73)  1.38 (0.30-11.30) 1.64 (0.30-72.98)  0.61 (0.57-49.74)  1.32 (0.24-19.36) 2.13 (0.22-72.98)  0.77 (0.31-49.74)  1.07 (0.22-19.36) 0.33 (0.22-0.43) 2.21 (0.29-72.98) 3.21 (0.24-10.74) | 0.36  0.28  0.86  0.66 0.68  0.97  0.95 0.94  1.0  0.57 0.40  0.47 0.95 - **0.039** 0.17 |

**Supplementary Table S1. E1 and E2 concentrations in primary cancerous and histopathologically unchanged tissue samples from patients with CRC.**
