# Supplementary material for: Significance of intratissue estrogen concentration coupled with estrogen receptors levels in colorectal cancer prognosis
Source: Oncotarget. 2017 Dec 14;8(70):115546–60. doi: 10.18632/oncotarget.23309 (PMC5777792; doi:10.18632/oncotarget.23309)
Supplement: Supplementary file 3 [file oncotarget-08-115546-s003.doc]

| **ESR1** | **Primary cancerous tissue** | **Histopathologically unchanged tissue** |
| --- | --- | --- |
| **Age (years)**  **Gender**  **Localization**  **Histologic grade**  **TNM classification** | 0.25  0.14  0.85  0.43  0.14 | 0.66  0.83  1.00  0.91  0.84 |

| **ESR2** | **Primary cancerous tissue** | **Histopathologically unchanged tissue** |
| --- | --- | --- |
| **Age (years)**  **Gender**  **Localization**  **Histologic grade**  **TNM classification** | 0.77  0.11  0.44  0.43  0.14 | 0.42  0.30  0.13  0.61  0.08 |

| **E1** | **Primary cancerous tissue** | **Histopathologically unchanged tissue** |
| --- | --- | --- |
| **Age (years)**  **Gender**  **Localization**  **Histologic grade**  **TNM classification** | **0.048**  0.88  0.39  0.41  0.39 | 0.16  0.92  0.19  0.87  0.37 |

| **E2** | **Primary cancerous tissue** | **Histopathologically unchanged tissue** |
| --- | --- | --- |
| **Age (years)**  **Gender**  **Localization**  **Histologic grade**  **TNM classification** | 0.59  0.55  0.68  0.41  0.65 | 0.72  0.70  0.79  0.39  **0.031** |

| **E1 to E2 ratio** | **Primary cancerous tissue** | **Histopathologically unchanged tissue** |
| --- | --- | --- |
| **Age (years)**  **Gender**  **Localization**  **Histologic grade**  **TNM classification** | 0.082  0.55  0.74  0.68  0.74 | 0.14  0.93  0.91  0.27  0.48 |

**Supplementary Table S2. E1 and E2 concentrations, E1 to E2 ratio and ESR1 and ESR2 transcript levels in different categorical groups within cancerous or histopathologically unchanged tissue of patient with CRC.**
