# Supplementary material for: Significance of intratissue estrogen concentration coupled with estrogen receptors levels in colorectal cancer prognosis
Source: Oncotarget. 2017 Dec 14;8(70):115546–60. doi: 10.18632/oncotarget.23309 (PMC5777792; doi:10.18632/oncotarget.23309)
Supplement: Supplementary file 4 [file oncotarget-08-115546-s004.doc]

| **ESR1** | **Primary cancerous tissue** | **Histopathologically unchanged tissue** | **p^a^** |
| --- | --- | --- | --- |
|  | median (range) | |  |
| **Age (years)**  <60 >60  **Gender**  Female  Male  **Localization**  Proximal colon  Distal colon  Rectum  **Histologic grade**  G1  G2  G3  **TNM classification**  I  IIA IIC IIIA IIIB IIIC | -0.29 (-3.78-4.61)  -0.66 (-3.78-3.69) -0.29 (-3.10-4.61)  -0.011 (-3.78-3.17)  -0.75 (-3.10-4.61)  -0.51 (-2.95-4.61)  -0.87 (-3.78-1.97) -0.34 (-3.10-3.69)  -0.024 (-3.10-3.17)  -0.51 (-3.78-3.69) -0.024 (-3.10-3.17)  -0.29 (-2.93-1.31)  -0.81 (-3.78-3.69) 0.40 (0.31-0.49) 0.83 (0.10-1.16) -0.48 (-3.10-3.17) 0.43 (-2.58-3.37) | 1.35 (-4.99-4.14)  1.01 (-1.59-2.75) 1.35 (-4.99-4.14)  1.32 (-1.59-3.38)  1.40 (-4.99-4.14)  1.37 (-4.99-4.14)  1.32 (-1.59-2.74) 1.35 (-1.27-3.57)  0.55 (-1.28-2.90)  1.29 (-4.99-4.14) 1.33 (-0.076-3.57)  1.02 (-1.27-2.90)  1.33 (-4.99-3.39) 1.39 (1.28-1.51) 2.27 (1.32-2.60) 1.12 (-0.99-3.57) 1.52 (-0.04-2.74) | < 0.0001  0.00083 < 0.0001  0.000126  <0.0001  0.000044  0.000532 0.000002  0.12  <0.0001 0.000659  0.024  0.000002  - - 0.0003 0.43 |

| **ESR2** | **Primary cancerous tissue** | **Histopathologically unchanged tissue** | **p^a^** |
| --- | --- | --- | --- |
|  | median (range) | |  |
| **Age (years)**  <60 >60  **Gender**  Female  Male  **Localization**  Proximal colon  Distal colon  Rectum  **Histologic grade**  G1  G2  G3  **TNM classification**  I  IIA IIC IIIA IIIB IIIC | -0.73 (-7.13-2.34)    0.11 (-3.84-1.84)  -0.73 (-7.13-2.34)  -0.48 (-7.13-2.34) -0.90 (1.98-5.39)  -0.84 (-7.13-1.98)  -1.20 (-3.62-0.25) -0.55 (-4.60-2.34)  -1.25 (-3.23-1.35)  -0.76 (-7.13-2.34) -0.37 (-4.52-1.71)  -0.64 (-3.41-1.52) -1.16 (-7.13-1.84) -1.36 (-3.01-0.28) 0.098 (-0.58-2.33) -0.41 (-4.60-1.84) -0.31 (-2.83-0.25) | 1.73 (-2.15-4.07)    1.78 (-1.25-2.59)  1.72 (-2.15-4.07)  1.79 (-1.25-2.95) 1.69 (-2.15-4.07)  1.93 (-2.15-3.07)  1.53 (-1.25-3.29) 1.77 (-1.71-4.07)  1.73 (-1.71-2.36)  1.76 (-2.15-4.07) 1.61 (0.026-3.07)  1.43 (-1.71-2.52) 1.83 (-2.15-3.07) 1.44 (0.78-2.11) 1.92 (0.18-2.07) 1.45 (-0.33-4.07) 1.92 (0.026-3.29) | 0.000001  0.00035  0.000001  0.000001 0.000001  0.000001  0.000002 0.000001  0.12  0.000001 0.000001  0.0037 0.000001 - - 0.000003 0.0011 |

**Supplementary Table S4. ESR1 and ESR2 transcript levels in primary cancerous and histopathologically unchanged tissue samples from patients with CRC.**
